# Residential radon exposure and brain cancer: an ecological study in a radon prone area (Galicia, Spain)

**DOI:** 10.1038/s41598-017-03938-9

**Published:** 2017-06-15

**Authors:** Alberto Ruano-Ravina, Nuria Aragonés, Karl T. Kelsey, Mónica Pérez-Ríos, María Piñeiro-Lamas, Gonzalo López-Abente, Juan M. Barros-Dios

**Affiliations:** 1 Department of Preventive Medicine and Public Health, University of Santiago de Compostela, Santiago, Spain; 2 CIBER de Epidemiología y Salud Pública, CIBERESP, Santiago, Spain; 3 Department of Epidemiology, Brown School of Public Health, Providence, Rhode, Island USA; 4 Environmental and Cancer Epidemiology Unit, National Centre for Epidemiology, Carlos III Institute of Public Health, Madrid, Spain; 5 Preventive Medicine Unit, Santiago de Compostela Clinic University Hospital, Santiago, Spain

## Abstract

We aimed to know if radon concentration is associated with municipal mortality due to brain cancer in Galicia, Spain. We designed an ecological study taking as study unit Galician municipalities. To be included, municipalities had to have at least three radon measurements. We correlated radon concentrations with municipal mortality due to these malignant tumors during the period 1999–2008. We calculated the relative risk of dying of brain cancers for each municipality and correlated this value with municipal radon concentration using Spearman’s Rho. 251 municipalities were included, with close to 3,500 radon measurements and an average of 14 radon measurements at each municipality. We observed a significant correlation between residential radon with brain cancer mortality for males and females and the intensity of the correlation was higher for females. These results were reinforced when the analysis was restricted to municipalities with more than 5 radon measurements: Spearman’s Rho 0.286 (p-value < 0.001) and Spearman’s Rho 0.509 (p-value < 0.001) for males and females, respectively. These results suggest an association between residential radon and brain cancer mortality. More research using more robust epidemiological designs is needed to confirm these findings.

## Introduction

Brain cancers are not frequent. Age adjusted incidence rates for Europe are 6.3 and 4.7 cases per 100,000 inhabitants for males and females, respectively^1^, while in Spain these rates are 6.1 and 4.2^1^. Despite its low incidence, these tumors have a high mortality, with a 5-year survival of only 22%^2^. Differences on survival are minimum by sex. These tumors can be divided according to their histology, and they can be classified as gliomas, neuronal tumors, poorly differentiated tumors, meningiomas and other tumors.

There is poor information regarding the risk factors of brain tumors. Due to its low incidence it is difficult to assess the role of environmental and genetic factors, and also the possible interactions among them. Some hereditary syndromes increase the risk of these tumors, such as such as neurofibromatosis, Li-Fraumeni syndrome, tuberous sclerosis, Von Hippel-Lindau syndrome or retinoblastoma^3, 4^.

One of the most established risk factors for brain cancers is exposure to ionizing radiation, mainly from medical imaging^5^. A recent study has observed an association between computerized tomographies and brain cancers^6^. Though some researchers have suggested that exposure to electromagnetic radiation could be associated with brain cancers, this relationship has not been confirmed^4^. Something similar happened with the electromagnetic radiation coming from the use of cell phones, but the INTERPHONE study did not find a consistent association^7^. Tobacco and alcohol consumption have not been associated with these cancers^8^.

Radon is a naturally occurring gas that is released by the bedrocks present in the earth crust. It is odorless, colorless and tasteless and it is settled in the Uranium 238 decay chain. It has a half-life of 3.8 days and it is not a health problem by itself. The real problem comes from its short-lived descendants Polonium 214 and Polonium 218, which have a very short half-life. When these elements disintegrate, they release alpha particles that impact cells located in the lung epithelium and finally can cause lung cancer^9^. Radon was declared a human carcinogen by the International Agency for Research on Cancer (IARC) in 1988^10^. Radon accumulates indoors in houses and workplaces, and it can entry from the soil mainly through cracks and joints and it is also highly soluble in water. It is accepted that people living in places with high radon concentrations have a higher risk of lung cancer^9^.

Since brain cancer is associated with ionizing radiation^11^, it is biologically plausible that residential radon exposure might increase its risk. Organs apart the lungs may receive low doses from radon exposure, as stated in the WHO report on indoor radon^9^. Nevertheless, very few studies have assessed the relationship between radon and brain tumors. Radiation dose received by the brain due to radon and its byproducts is much lower than that received by other organs^12^. After one year of an average exposure of 200 Bq/m^3^ by inhalation, the brain would receive between 0.06 and 0.15 mSv whereas the lung in the same situation would receive between 35.8 and 159 mSv. Since 200 Bq/m^3^ is approximately equivalent to 10 mSV per year (one computed tomography), the brain would receive a dose equivalent to 20 computed tomographies during 20 years. It has been proposed that macrophages might phagocyte small solid particles in the lungs from radon descendants that could reach the brain through the blood^12^. The existing literature is controversial and most studies have been performed in miners. In general adult population, a Danish study observed a statistically significant association between residential radon and brain cancers^13^ but a study based on the Cancer Prevention Study-II showed no association between mean county radon levels and brain cancer mortality^14^.

Galicia, the study area, has been defined previously as a radon prone region. Approximately 20% of all dwellings have radon concentrations above the Environmental Protection Agency action level (148 Bq/m^3^)^15–17^. The high radon concentrations found in Galicia facilitate the analysis of possible associations between radon and other cancers different than lung cancer^18^ because it is easier to have municipalities with high radon concentrations and correlate if these municipalities have higher or lower brain cancer mortality.

The aim of the present study is to analyze if there is a correlation between residential radon levels and brain cancer mortality at a municipal level in Galician population. A subanalysis will be performed to know if this correlation differs by sex.

## Methods

### Setting and study design

The study has been performed in Galicia, Northwest of Spain. Galicia limits to the North and West with the Atlantic Ocean, to the South with Portugal and to the East with mainland Spain. Around half of the population lives in rural areas, mainly in detached houses.

We designed an ecological study aimed to analyze the correlation between residential radon and risk ratios of brain cancer mortality for Galician municipalities. We established as inclusion criterion that each municipality had to have at least 3 residential radon measurements.

### Radon measurements

Radon measurements were obtained from the Galician Radon Map and from controls belonging to two case-control studies performed previously^17, 19^. The Galician Radon Map has been developed by the University of Santiago de Compostela to identify those areas with the highest radon concentrations in Galicia. Participants in the Galician Radon Map were selected from the general population through a stratified random sampling weighted by the population of each municipality^16^. The sampling was designed so that at least two radon measurements were available in those municipalities less populated.

Controls from two hospital-based case-control studies were also included in the present research. This inclusion allowed us to increase the sample size in more than 600 radon measurements. Controls from one case-control study^17^ were recruited from two Galician hospitals among people residing in municipalities covered by those hospitals. The second case-control study had a similar design^19^, with the difference that it recruited controls living in the catchment areas of four different Galician hospitals. We did not include lung cancer cases since radon is associated with lung cancer and radon concentration in cases could bias our radon measurements towards a higher median.

For all measurements, radon detectors were placed for a minimum of three months and a maximum of six. We used alpha-track detectors (Radosys Inc, Budapest, Hungary). Radon devices were placed by a radon technician or by participants in the main bedroom and, in some cases, in the living room of the measured dwelling. Detectors were away from doors, windows or electric devices, and at a distance to the floor between 60 and 180 cm. When the measurement period finished, the detectors were sent by the participants in a specially sealed envelope to the Galician Radon Laboratory, located at the Santiago de Compostela Clinic University Hospital. The Galician Radon Laboratory regularly takes part in quality control procedures organized by the University of Cantabria and the Nuclear Safety Council of Spain with excellent results^20, 21^. In some dwellings, we included double measurements to check the reliability of our processes. All radon measurements were adjusted taking into account seasonal variations.

### Brain cancer mortality

We calculated standardized mortality ratios (SMR) for brain cancer for all Galician municipalities for a 10-year period (1999–2008) using data supplied by the National Statistics Institute. We used all Spanish death entries for the period considered corresponding to brain cancer (International Classification of Diseases[ICD], 9th revision code 191, ICD-10 code C71) broken down by sex, age group (18 groups), five-year period (1999–2003, 2004–2008) and municipality. The municipal populations, also broken down by sex and age group, were drawn from the 2001 census and 2006 municipal roll. These years correspond to the midway points of the two quinquennia that comprise the study period. Person-years for each five-year period were estimated by multiplying these populations by 5.

Firstly, standardized mortality ratios (SMR) were computed as the ratio of observed versus expected deaths. Expected cases were computed taking Spanish brain cancer mortality rates as reference, broken down by sex, age and quinquenium. In a second step, smoothed municipal relative risks (RR) were calculated using the conditional autoregressive spatial model developed by Besag, York and Mollié (BYM)^22^. It is a spatial Poisson model, with observed cases as the dependent variable, expected cases as offset, and two types of random-effects terms that take the following into account: (a) the effects which vary in a structured manner in space (municipal contiguity); and, (b) a component that models the effects which vary among municipalities in an unstructured manner (municipal heterogeneity). The criterion of contiguity used was adjacency of municipal boundaries. The model takes the following form:

$$\begin{array}{l}{O}_{i}\sim Po({E}_{i}{\lambda }_{i})\\ \mathrm{log}({\lambda }_{i})=\alpha +{h}_{i}+{b}_{i}\end{array}$$

where: *λ*_i_ is the relative risk in area i; O_i_ is the number of deaths in area i; P_o_ is Poisson distribution; α is the intercept; E_i_ are the expected number of cases; h_i_ is the municipal heterogeneity term; and b_i_ is the spatial term. Integrated nested Laplace approximations (INLAs)^23^ were used as a tool for Bayesian inference. For this purpose, we used R-INLA. Municipal relative risks (RR) have been independently estimated in both men and women.

### Statistical analysis

We performed a descriptive analysis (univariate) on residential radon measurements and also on brain cancer mortality RR broken down by municipality. For each municipality with more than 3 radon measurements we obtained the number of total measurements and the median residential radon. We obtained the mortality risk of brain cancer separately by males and females. The descriptive analysis was followed by a bivariate analysis where we used Spearman correlations to associate median municipal radon concentration with brain cancer mortality RR for males and females, respectively. We also displayed graphically the correlations comparing relative risks with residential radon levels. We also performed a sensitivity analysis including only those municipalities having more than 5 radon measurements. We considered statistical significance when p-value was lower than 0.05. The analysis was performed with IBM SPSS v20 (IBM, Armonk, NY, USA).

## Results

This study has included 3,498 radon measurements in 251 Galician municipalities, an average of 14 measurements per municipality. Sixty-four municipalities were not included (18.7% of the total) because they had less than 3 radon measurements. 50.6% of the included municipalities had radon levels (geometric mean) above WHO recommendation (100 Bq/m^3^), 25.9% had geometric means above EPA action levels (148 Bq/m^3^) and 13.5% had geometric means above 200 Bq/m^3^. A description of radon concentrations by municipality can be observed in Table 1.

**Table 1:** Characteristics of residential radon measurements and risk of brain cancer in the included municipalities.

Variables	N (%)
Number of radon measurements by municipality	
3–4	78 (31.1)
5–14	120 (47.8)
15–29	32 (12.7)
30–49	10 (4.0)
≥50	11 (4.4)
Radon concentration by municipality (Geometric mean Bq/m^3^)	
<100	124 (49.4)
100–147	62 (24.7)
148–199	31 (12.3)
200–249	20 (8.0)
≥250	14 (5.6)
Relative Risk of brain cancer in males by municipality	
<1, 0	8 (3.2)
1, 0–1, 04	136 (54.1)
1, 05–1, 09	84 (33.5)
≥1, 10	23 (9.2)
Relative Risk of brain cancer in females by municipality	
<1, 0	151 (60.2)
1, 0–1, 04	53 (21.1)
1, 05–1, 09	29 (11.5)
≥1, 10	18 (7.1)

Regarding mortality due to brain cancer during the period 1999–2008, there were 949 overall deaths among males and 758 among females in the included municipalities. The range of deaths due to brain cancer (maximum-minimum) for males was 0–99 and 0–107 for females. For males, most municipalities (136, 54.1%) had a low relative risk of death for these cancers (between 1 and 1.04). Only 23 (9.2%) of municipalities had a relative risk of death for males higher than 1.10. For females, the risk of death for these cancers was lower compared to males. Only 18 municipalities (7.1%) had a relative risk of death higher than 1.10. This information is shown in Table 1.

**Figure 1:**
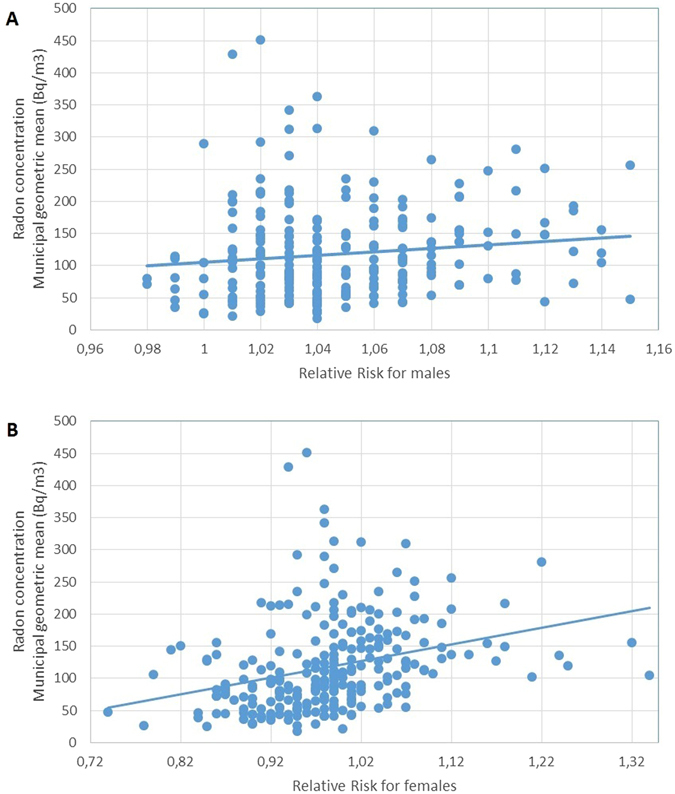
Scatterplot showing the correlation between municipal radon concentration and relative risk of mortality for brain cancer in (**A**) males, and (**B**) females.

Regarding the correlation between residential radon and brain cancer mortality, it was statistically significant for males and females, for females the association observed was also moderate. The Spearman’s Rho was 0.164 (p-value 0.009) for males and 0.433 (p-value < 0.001) for females. When the analysis was restricted to those municipalities having at least 5 or more radon measurements the statistical significance persists and the strength of the association increases (measured through the Spearman’s Rho). For males, Spearman’s Rho was 0.286 (p-value < 0.001) and for females Spearman’s Rho was 0.509 (p < 0.001). The scatterplot showing the correlation between residential radon and brain cancer mortality can be observed in Fig. 1A and B. For both sexes the correlation is clear. If we analyze only those municipalities with 10 or more radon measurements (84 of the 251, 26.6% of the total included), the correlation persists. For males, Spearman’s Rho is 0.314 (p = 0.004) and for females Spearman’s Rho is 0.642 (p-value < 0.0001).

Table 2 shows the 15 municipalities with the highest residential radon concentrations in Galicia with their relative risk (and posterior probability that RR > 1) of mortality for brain cancers. It can be observed that for males, all municipalities have relative risks above one and 5 municipalities have RR above percentile 75. For females, 6 municipalities have relative risks above percentile 75 of all relative risks of the municipalities included in the analysis (n = 251), though the remaining 9 municipalities had relative risks below 1 (but very close to 1).

**Table 2:** Municipalities with the ranked by residential radon concentration and relative risk for brain cancer in males and females.

Municipality	Median radon concentration (Bq/m^3^)	RR in men^a^	PP RR >1^c^	RR in women^b^	PP RR > 1^c^
O Bolo	450 (n = 3)	1.02	0.56	0.96	0.39
A Mezquita	429 (n = 3)	1.01	0.52	0.94	0.35
Padrenda	363 (n = 5)	1.04	0.61	0.98	0.45
A Pobra de Trives	341 (n = 3)	1.03	0.60	0.98	0.43
Esgos	313 (n = 3)	1.04	0.61	0.99	0.47
Cenlle	312 (n = 5)	1.03	0.60	**1.02**	0.55
Fornelos de Montes	309 (n = 3)	1.06	0.68	**1.07**	0.69
Samos	292 (n = 3)	1.02	0.57	0.95	0.34
Cualedro	289 (n = 4)	1.00	0.51	0.98	0.45
Mos	281 (n = 20)	**1.11**	0.79	**1.22**	0.91
Sober	271 (n = 3)	1.03	0.61	0.99	0.47
Campo Lameiro	264 (n = 3)	**1.08**	0.73	**1.06**	0.68
Salceda de Caselas	255 (n = 5)	**1.15**	0.86	**1.12**	0.78
Meaño	251 (n = 8)	**1.12**	0.80	**1.08**	0.69
Bueu	247 (n = 8)	**1.10**	0.73	0.98	0.45

^a^In bold those municipalities with relative risk of brain cancer above percentile 75 (1.07).

^b^In bold those municipalities with relative risk of brain cancer above percentile 75 (1.02).

^c^Posterior probability that RR >1.

## Discussion

There is very little information available on the possible relationship between residential radon and brain cancer. In the present study, we have observed a statistically significant correlation between residential radon exposure at a municipal level and mortality due to brain cancer for males and females. The association appears to be slightly higher for females. The correlation increases when the analysis is restricted to municipalities having 5 or more radon measurements and is clearly identifiable in the scatterplots. This is the second study performed in Europe on this topic and the first performed in a high-risk radon area.

The possible relationship between indoor radon and brain cancer in adult population has been studied in three different investigations. The study by Turner *et al*. had a cohort design using the cancer prevention Study II cohort^14^. The study analyzed brain tumors mortality, and radon measurements were estimated assuming the median of each county where each participant resided. In this ecological study including 2,232 cases, each county had an average of 10 radon measurements, and no effect was observed for residential radon exposure, with a Hazard Ratio of HR 0.98 per 100 Bq/m^3^ (95% CI 0.83–1.15). Of note, Turner *et al*. used counties as the geographical units of study, which have much higher area and population than Galician municipalities. Therefore, in relative terms, the present study has a higher number of radon measurements to characterize municipalities. Furthermore, while in the Turner *et al*. study the average mean concentration of radon was 53.5 Bq/m^3^, in our study only 43 of the 251 studied municipalities (17.1%) had residential radon concentrations below the Turner’s average. A second study was performed in Denmark by Bräuner *et al*., where the authors observed a statistically significant effect of 1.44 (95% CI 1.07–1.93) for each 100 Bq/m^3^ of increment in residential radon concentration^13^. In this study, radon concentration was estimated in each participant residence, using a validated method. The remaining study, published in 1993 and performed in Maine (USA), analyzed the presence of radon in water and did not find any association with brain tumors^24^ using an ecological design.

There is more published literature in miners analyzing radon exposure and brain cancers, though the results are not consistent. Results from the French Miners’ Cohort^25–27^ suggested an association with radon exposure, while results from the German Miners’ cohort (Wismut Mining Company) did not^28, 29^. Other miners’ studies^30, 31^ have not observed any association while other observed a close to significant association^32^. Only one of these studies has reached statistical significance, with observed to expected cases ratio of 2.00 (95% CI 1.09–3.35)^27^. Most of these studies had a poor adjustment for covariates and given the low frequency of brain tumors, the number of observed cases was very low in most of them, causing imprecise estimations. Furthermore, though some studies adjusted by other carcinogens that can be present in mines (such as gamma rays, arsenic or dusts), residual confounding could not be excluded.

There is biological plausibility to support an association between residential radon and brain cancer. Though the effective dose reaching the brain from radon exposure is much lower than that received by the lungs, it has been estimated that brain would receive a dose between 0.06 and 0.15 mSv after one year of an average exposure by inhalation of 200 Bq/m^3^. In the studied geographical area, 34 municipalities have a geometric mean of residential radon concentration higher than 200 Bq/m^3^. If we consider that Galician population tends to live in the same house for decades^19^, we could estimate that part of our population is receiving important amounts of radiation due to residential radon exposure and therefore could promote brain cancer. A strong relationship between residential radon and lung cancer has already been observed in our region in ever and never-smokers^17, 19^. Few studies have assessed the biological effect of radon in the brain. There is a research on how low radon concentrations could activate superoxide dismutase in rats but high levels or long exposures inactivate it^33^. Anyway, this observation does not explain a possible carcinogenic effect.

The higher correlation observed for women compared to men might be due to a more intense exposure to radon among females. Older cohorts of Galician women have been predominantly housewives and women have started working out of their homes in the last two decades. This means that older women were more exposed to residential radon than their male counterparts due to this more intense exposure at home. A higher susceptibility for women to alpha radiation cannot be disregarded either.

Our results should be interpreted taking into account some limitations. Regarding brain cancer data, since there is not a population-based cancer incidence registry in Galicia, mortality is the only available source of information. While due to its low survival (5-year relative survival is approximately 20% for malignant tumors)^34^, brain cancer mortality can be considered a good estimator of incidence data, a limitation of mortality information is that it didn’t allow us to analyze brain cancer by subtype, and we cannot exclude that radon exposure may have a particular impact in some morphological types.

As regards to the methodology, an obvious limitation of this ecological study is that causal associations cannot be established. We cannot disregard that some unmeasured or unknown risk factors for brain tumors jeopardize the association observed. In any case, we do not consider that residential radon is associated with other risk factors for brain tumors, as has been observed in other studies.

Radon exposure measurement may also be a concern. In the present study, we have a limited number of radon measurements in some municipalities, but some of them have a small population (in many cases below 500 inhabitants), and 3 measurements could be considered an acceptable number to estimate indoor radon in such small areas. Altogether, we believe that the available radon measurements are adequate to characterize average residential radon, as has been done in other similar studies^14^.

A further limitation is the use of SMR, which has long been criticized since it can be misused and misinterpreted, due to the intrinsic difficulty of comparison among SMRs, because the weighting of each SMR depends on the age structure of the study population^35, 36^. Though some authors have proposed special solutions^37^, the use of SMRs has become widespread due to its easy interpretability and because when viewed as a weighted average of the ratios of age-specific rates for cohort and standard population, the weights minimize the variance of weighted average^36^. In practical terms, the SMR tend to be less sensitive to numerical instabilities in one or two of the age-specific rates. Some problems of the SMR are extensive to the use of age-specific rates, since in both cases some information is lost. In our experience and with Galician data, the inclusion of the age component (with person-years as offset) in the BYM models for smoothing RRs does not alter the results substantially.

Finally, concerning statistical modelling, we used the BYM model to obtain a mortality indicator which avoids the problem of the small area analysis, allowing us to address the correlation study, though we cannot discard completely that the spatial dependence was not the same throughout the territory of Galicia. The model has to conceal SMRs with residential radon concentration at a municipal level.

There are no other relevant sources of ionizing radiation exposure apart from radon in the study setting. Radon exposure comprises approximately 50% of all lifetime exposure to ionizing radiation, followed by medical exposure (15–20%). Given that the study area is a radon prone-area we could say that radon exposure probably accounts for more than a 50%. Since Galician population is covered by the same health provider (Galician Health Service), which is universal and free, we do not expect relevant differences in ionizing exposure coming from medical devices for the population residing in each of the included municipalities.

The present study has also some advantages. The first one is that Galicia is a radon prone area, as has been published before^15, 16^. In fact, of all the available studies on this topic, Galicia is the area which has the highest residential radon levels. This characteristic makes easier to analyze the effect of radon in brain cancer mortality. The second advantage of this work is that Galician population tends to live for a long time in the same dwelling and therefore the potential latency period for radon exposure could be fulfilled more easily when compared to other geographical settings where people has more mobility. A third advantage is that we are using real radon measurements instead of estimations of radon concentration, as has been done in other investigations^13^. This means that the characterization of radon exposure is more reliable in the present study. A last advantage is the use of BYM model, which is a suitable tool for obtaining smoothed indicators in spatial epidemiology. In our context, it has been used to obtain a mortality indicator which bypasses the problem of the “small area” analysis, allowing us to address the correlation study.

To conclude, this study suggests that a possible association between residential radon and mortality due to brain cancer might exist. This association is biologically plausible due to the role of ionizing radiation from other sources on the onset of these malignant tumors. To have a deeper knowledge of this relationship, it is important to perform well-designed case-control studies, preferably in radon prone areas to shed more light on this association. Health authorities should promote these studies and raise public awareness on the already known health risks of indoor radon exposure.

## References

[1] Ferlay J. *et al*. *GLOBOCAN 2012 v1.0, Cancer Incidence and Mortality Worldwide: IARC CancerBase No. 11 [Internet]*. (International Agency for Research on Cancer).

[2] De Angelis R (2014). Cancer survival in Europe 1999–2007 by country and age: results of EUROCARE–5-a population-based study. Lancet Oncol..

[3] Ranger AM, Patel YK, Chaudhary N, Anantha RV (2014). Familial syndromes associated with intracranial tumours: a review. Childs Nerv. Syst. ChNS Off. J. Int. Soc. Pediatr. Neurosurg..

[4] Goodenberger ML, Jenkins RB (2012). Genetics of adult glioma. Cancer Genet.

[5] Bondy ML (2008). Brain tumor epidemiology: consensus from the Brain Tumor Epidemiology Consortium. Cancer.

[6] Mathews JD (2013). Cancer risk in 680,000 people exposed to computed tomography scans in childhood or adolescence: data linkage study of 11 million Australians. BMJ.

[7] Interphone Study Group. Brain tumour risk in relation to mobile telephone use: results of the INTERPHONE international case-control study. *Int*. *J*. *Epidemiol*. **39**, 675–694 (2010). 10.1093/ije/dyq079 20483835

[8] U.S. Department of Health and Human Services. *The Health Consequences of Smoking—50 Years of Progress: A Report of the Surgeon General*. (Centers for Disease Control and Prevention, National Center for Chronic Disease Prevention and Health Promotion, Office on Smoking and Health 2014).

[9] *WHO handbook on indoor radon: a public health perspective*. http://apps.who.int/iris/bitstream/10665/44149/1/9789241547673_eng.pdf Date of access: 01/04/2017 (World Health Organization 2009). 23762967

[10] *Man made mineral fibres and radon:*… *views and experts opinions of an IARC Working Group on the Evaluation of Carcinogenic Risks to Humans*, *which met in Lyon 16*–*23 June 1987* (1988).

[11] *IARC monographs on the evaluation of carcinogenic risks to humans*, *volume 100 D*, *radiation: this publication represents the views and expert opinions of an IARC Working Group on the Evaluation of Carcinogenic Risks to Humans*, *which met in Lyon*, *02–09 June 2009* (IARC, 2012).

[12] Kendall GM, Smith TJ (2002). Doses to organs and tissues from radon and its decay products. J. Radiol. Prot. Off. J. Soc. Radiol. Prot..

[13] Bräuner EV (2013). Residential radon and brain tumour incidence in a Danish cohort. PloS One.

[14] Turner MC (2012). Radon and nonrespiratory mortality in the American Cancer Society cohort. Am. J. Epidemiol.

[15] Barros-Dios JM, Barreiro MA, Ruano-Ravina A, Figueiras A (2002). Exposure to residential radon and lung cancer in Spain: a population-based case-control study. Am. J. Epidemiol..

[16] Barros-Dios JM, Ruano-Ravina A, Gastelu-Iturri J, Figueiras A (2007). Factors underlying residential radon concentration: results from Galicia, Spain. Environ. Res..

[17] Barros-Dios JM (2012). Residential radon exposure, histologic types, and lung cancer risk. A case-control study in Galicia, Spain. Cancer Epidemiol. Biomark. Prev. Publ. Am. Assoc. Cancer Res. Cosponsored Am. Soc. Prev. Oncol..

[18] Ruano-Ravina A, Aragonés N, Pérez-Ríos M, López-Abente G, Barros-Dios JM (2014). Residential radon exposure and esophageal cancer. An ecological study from an area with high indoor radon concentration (Galicia, Spain). Int. J. Radiat. Biol..

[19] Torres-Durán M (2014). Lung cancer in never-smokers: a case-control study in a radon-prone area (Galicia, Spain). Eur. Respir. J..

[20] Vargas A, Ortega X (2007). Influence of environmental changes on integrating radon detectors: results of an intercomparison exercise. Radiat. Prot. Dosimetry.

[21] Gutierrez-Villanueva JL (2013). Intercomparison exercise on external gamma dose rate under field conditions at the laboratory of natural radiation (Saelices el Chico, Spain). Radiat. Prot. Dosimetry.

[22] Besag, J., York, J. Mollie, A. Bayesian image restoration, with two applications in spatial statistics. **43**, 1–59 (1991).

[23] Rue H, Martino S, Chopin N (2009). Approximate Bayesian inference for latent Gaussian models by using integrated nested Laplace approximations. J. R. Stat. Soc. Ser. B Stat. Methodol..

[24] Hess CT, Weiffenbach CV, Norton SA (1983). Environmental radon and cancer correlations in Maine. Health Phys..

[25] Tirmarche M, Raphalen A, Allin F, Chameaud J, Bredon P (1993). Mortality of a cohort of French uranium miners exposed to relatively low radon concentrations. Br. J. Cancer.

[26] Vacquier B (2008). Mortality risk in the French cohort of uranium miners: extended follow-up 1946-1999. Occup. Environ. Med..

[27] Vacquier B (2011). The influence of multiple types of occupational exposure to radon, gamma rays and long-lived radionuclides on mortality risk in the French ‘post-55’ sub-cohort of uranium miners: 1956–1999. Radiat. Res..

[28] Kreuzer M, Walsh L, Schnelzer M, Tschense A, Grosche B (2008). Radon and risk of extrapulmonary cancers: results of the German uranium miners’ cohort study, 1960–2003. Br. J. Cancer.

[29] Kreuzer M (2010). Radon and risk of death from cancer and cardiovascular diseases in the German uranium miners cohort study: follow-up 1946–2003. Radiat. Environ. Biophys..

[30] Schubauer-Berigan MK, Daniels RD, Pinkerton LE (2009). Radon exposure and mortality among white and American Indian uranium miners: an update of the Colorado Plateau cohort. Am. J. Epidemiol..

[31] Darby SC (1995). Radon and cancers other than lung cancer in underground miners: a collaborative analysis of 11 studies. J. Natl. Cancer Inst..

[32] Darby SC, Radford EP, Whitley E (1995). Radon exposure and cancers other than lung cancer in Swedish iron miners. Environ. Health Perspect..

[33] Ma J, Yonehara H, Ikebuchi M, Aoyama T (1996). Effect of radon exposure on superoxide dismutase (SOD) activity in rats. J. Radiat. Res. (Tokyo).

[34] Sant M (2012). Survival of European patients with central nervous system tumors. Int. J. Cancer.

[35] Rothman, K. J. *Modern epidemiology*. (Little, Brown 1986).

[36] Breslow, N. E. & Day, N. E. Statistical methods in cancer research. Bd. 2: The design and analysis of cohort studies. (International Agency for Research on Cancer 1987). 3329634

[37] Rosenbaum PR, Rubin DB (1984). Difficulties with regression analyses of age-adjusted rates. Biometrics.

